# The Prevalence of Tuberculosis

**Published:** 1891-06

**Authors:** Daniel D. Lee

**Affiliations:** Instructor in Anatomy, Veterinary Dept, Harvard University


### THE PREVALENCE OF TUBERCULOSIS.

By Daniel D. Lee, M. D. V.

*Instructor in Anatomy, Veterinary Dept, Harvard University.*

In the March number of this Journal for the year 1890, I published an article on the Present Attitude of Veterinarians on the Subject of Tuberculosis, in which I took the ground that the present method of wholesale slaugter of all cattle that were tuberculous, was not only going to be ineffectual in stamping out the disease ; but that it was also an unjustifiable destruction of property, because no steps are taken to quarantine human beings suffering from the disease.

In the past year various wild statements have been made about the frightful prevalence of tuberculosis in American cattle; and these statements certainly tend to needlessly prejudice European countries against American beef.

The only statistics on tuberculosis that I know to have been compiled, and which include *different classes* of cattle, are those given in the report of the Board of Health of the City of Boston for 1890. These statistics are furnished by Dr. Alexander Burr, Inspector of Meat at the Brighton Abattoir. All the pathological specimens that he obtains are carefully examined by him, under the supervision of Dr. William F. Whitney, at the Harvard Medical School.

I give the tables below (that refer to tuberculosis):

| Class of Animal. | Number. | Tuberculus. | Per Cent. |
|---|---|---|---|
| (1) Whole number of all kinds of cattle........................ | 28,296 | 54 | 0.19 |
| (2) Cows from Eastern States..... | 1,153 | 52 | 4.5 |
| (3) Oxen........................ | | 1 | |
| (4) Western Cow...... ........ ... | | 1 | |
| (5) Old cows sent to dead-house, which have died in the city and its neighborhood....... | 116 | 12 | 10.3 |

From this table we find that the percentage of *all kinds* of cattle tuberculous is astoundingly small, $\frac{19}{100}$ per cent., less than a $\frac{1}{5}$ of one per cent. Next we find that one ox was tuberculous and only one Western cow. The Eastern cows $4\frac{1}{2}$ per cent.

Finally comes the last class of the cows coming from Boston and vicinity to the dead house. These cows belong to the class of "milk machines" that are said to be tuberculous as high as 35 per cent. and 50 per cent., and even in them we find it only $10\frac{3}{10}$ per cent.

Dr. Burr then calls attention to Dr. Michener's report in regard to the cows from about the city of New York. He finds in 5,000 cows, 1,379 unthrifty cows, of which 11 per cent. were tuberculous, and of the remainder four to five per cent. Now Dr. Burr and Dr. Michner both came very near each other in their estimate, and careful statistics of this sort are certainly of more value than statements of percentage based on the examinations of a few herds of "milk machines," for the reasons, first; that such cattle do not represent the condition of *all* the cattle in a State or Country; and secondly, that no statistics of the prevalence of tuberculosis are of the slightest value unless based on post mortem examinations, the diagnosis in life being often impossible as is well known.

The use of Koch's lymph as a diagnostic agent in bovine tuberculosis would of course make the diagnosis of the disease possible during life in doubtful cases, but unfortunately the reaction which follows the use of this material cannot be relied upon. This has been proved by its use on human beings. Numerous experiments have been made on cattle with Koch's lymph, in Europe, and it seems from the reports of these experiments that I have read, that the reaction is even more uncertain in cattle than in people.

It has been said that Dr. Burr's statistics are not to be taken as a fair average for the state, because "coughers" would not be sent to the abattoir, where they would have to be inspected, but to some sausage factory. I have a report from the same source extending from October, 1889, to April, 1890, which gives an account of the first six months of Dr. Burr's work, before the public became aware that a careful inspection was made at the abattoir. I give it below :

| | | |
|---|---|---|
| Total number of cows and steers killed, from October to April, - - - | 15,506 | |
| Percentage tuberculous, - - - | $\frac{17}{100}$ | per cent. |
| Eastern cows, tuberculous, - - - | $3\frac{3}{5}$ | " |

$\frac{17}{100}$ per cent is very near $\frac{19}{100}$ per cent of the last report, but a *little lower*, this does not look as if greater care was taken to send tuberculous cattle to other slaughtering establishments, and $3\frac{3}{5}$ per cent of Eastern cows is lower than the last report, which is $4\frac{1}{2}$ per cent.

The dead horse statistics are especially valuable. I give Dr. Burr's opinion in his own words.

"I may add in connection with the foregoing, that in connection with the Abattoir, we have an establishment where fertilizers are manufactured and dead animals of all kinds received, such as horses and cattle, many of which are cows; these animals represent a fair average of the cows of our neighborhood; having died, the owners have seldom any disposition to hide them. I have examined all the cattle brought here and so far, my record is as follows :"

| | |
|---|---|
| Received dead cows at Abattoir from Oct. 1, '89 till April 1, '90, - - - - | 80 |
| Number found with Tuberculous lesions, - - | 6 |
| Percentage, - - - - - | 7.5 |

No better opportunity, it seems to me, could be found to reach a fair average of the extent to which the disease prevails among our animals.

In this last table we find $7\frac{1}{2}$ per cent and $10\frac{3}{10}$ per cent in this year's report. Now all the percentages are larger for the past year than during the last six months. From them we can say that about $\frac{1}{5}$ of one per cent. of *American cattle* are tuberculous. That *milch cows*, being kept in confinement and therefore liable to tuberculosis, are affected as high as 4 or 5 per cent. That, unthrifty milch cows, used as "milk machines," badly fed or housed, run as high as 10 to 11 per cent. Don't let us forget that $\frac{1}{5}$ of one per cent. is the average for the country, this 2 in 1000. European statistics say 5 in 1000; we are better off than they are. To get a very high per cent. like 35 or 50 per cent. a report like this must be made.

"I examined a herd of sixty cows and found *four* cases of tuberculosis, *twenty* suspicious and *six* slightly suspicious, making in all 30 cases!" Now this is 50 per cent, but how many of these cows really have tuberculosis, perhaps five? Post mortem examinations are not made to back up such reports, and what are they worth?

